# Supplementary material for: Machine Learning Assisted Approach for Finding Novel High Activity Agonists of Human Ectopic Olfactory Receptors
Source: Int J Mol Sci. 2021 Oct 26;22(21):11546. doi: 10.3390/ijms222111546 (PMC8583936; doi:10.3390/ijms222111546)
Supplement: Supplementary file 1 [file ijms-22-11546-s001.zip › 1-Supporting_information_updated.pdf]

Supplementary Tables and Figures

Machine learning assisted approach for finding novel high activity agonists of human ectopic olfactory receptors

Amara Jabeen, [1] Claire A. de March, [2] Hiroaki Matsunami, [2] and Shoba Ranganathan*,[1]

[1] Department of Molecular Sciences, Macquarie University, Sydney, NSW 2109, Australia
[2] Department of Molecular Genetics and Microbiology, Duke University Medical Center, Durham, NC, 27710, USA
[3] Department of Neurobiology, Duke Institute for Brain Sciences, Duke University, Durham, NC 27710
* **Correspondence:** hiroaki.matsunami@duke.edu (H.M.); shoba.ranganathan@mq.edu.au (S.R.)

Table of Contents

Table S1: Experimentally tested odorants for OR1A1 2
Table S2: Experimentally tested odorants for OR2W1 12
Figure S1: Alignment of OR1A1, OR2W1 and 1U19 used for model building. 19
Figure S2: Diverse chemical nature of known agonists for (a) OR1A1 and (b) OR2W1 20
Figure S3: Workflow for feature selection for OR1A1 and OR2W1 21
Figure S4: Importance of selected features based on accuracy for (a) OR1A1 and (b) OR2W1 22
Table S3: Description and significance of selected features 23
Table S4: Statistical test for classifers performance 23
Figure S5: Search space statistics for (a) OR1A1 and (b) OR2W1 24
Figure S6: Alignment of olfactory receptor sequences with mutagenesis data and OR2W1.. 25
Figure S7: Cell surface expression of the studied ORs 26
Figure S8: Binding mode. 27
Figure S9: Interactions 28
References 29

Table S1: Experimentally tested odorants for OR1A1

Pubchem_CID	Odorant name	Class	Chemical nature
443157	(S)-(-)-citronellal[1]	Agonist	Aldehyde
61875	4-decenal[1]	Agonist	Aldehyde
7793	(S)-(-)-citronellol[1]	Agonist	Terpene
75427	(R)-(+)-citronellol[1]	Agonist	Terpene
439250	(S)-(-)-limonene[1]	Agonist	Terpene
5362695	Z-7-decanal[1]	Agonist	Aldehyde
5284507	Nerolidol[1]	Agonist	Alcohol
5702654	E-4-decanal[1]	Agonist	Aldehyde
643820	Nerol[1]	Agonist	Alcohol
64805	Helional[1]	Agonist	Aldehyde
11084068	(-)-Carveol[1]	Agonist	Alcohol
8878	Allyl heptanoate[2]	Agonist	Ester
31265	Ethyl hexanoate[3]	Agonist	Ester
439570	(-)-Carvone[3]	Agonist	Ketone
22227	(+)-Dihydrocarvone[3]	Agonist	Ketone
8174	1-decanol[2]	Agonist	Alcohol
8093	2-octanone[2]	Agonist	Ketone
7802	3-heptanone[2]	Agonist	Ketone
246728	3-octanone[2]	Agonist	Ketone
68110	4-chromanone[2]	Agonist	Ketone
15717	Allyl phenylacetate[2]	Agonist	Ester
3102	Benzophenone[2]	Agonist	Ketone
8785	Benzyl acetate[2]	Agonist	Ester
62378	Dihydrojasmone[2],[4]	Agonist	Ketone
15077	Nonanethiol[2]	Agonist	Sulphur compound
16724	(+)-Carvone[2]	Agonist	Ketone
61052	3-phenyl propyl propionate[5]	Agonist	Ester
92979	Androstadienone[5]	Agonist	Ketone
24433	Butyl anthranilate[5]	Agonist	Ester
637511	Cinnamaldehyde[5]	Agonist	Aldehyde
7047	Quinoline[5]	Agonist	Cyclic compound
440917	R-limonene[5]	Agonist	Terpene
6992244	(R/S)-octen-3-ol[3]	Agonist	Alcohol
16800	2-pentylpyridine[3]	Agonist	Cyclic compound
78126	2-phenylethanethiol[3]	Agonist	Sulphur compound
7654	2-Phenylethyl acetate[3]	Agonist	Ester
518810	3-Mercaptohexyl acetate[3]	Agonist	Ester
8815	Estragole[3]	Agonist	Cyclic compound
18686	Ethyl cyclohexanecarboxylate[3]	Agonist	Ester
637563	*Trans*-anethole[3]	Agonist	Cyclic compound

Pubchem_CID	Odorant name	Class	Chemical nature
529481	3-Methyl-2,4-nonanedione[3]	Agonist	Ketone
7590	Ethylphenyl acetate[3]	Agonist	Ester
7710	5-Pentyloxolan-2-one[3]	Agonist	Ketone
443159	(+)-Menthone[3]	Agonist	Ketone
62329	Musk xylol[6]	Agonist	Cyclic compound
66823518	Cosmone[6]	Agonist	Ketone
61585	Celestolide[6]	Agonist	Ketone
6997	2-ethylphenol[7]	Agonist	Cyclic compound
7128	Methyl isoeugenol[7]	Agonist	Cyclic compound
7685	P-Tolyl isobutyrate[7]	Agonist	Ester
7148	Propiophenone[7]	Agonist	Ketone
7888	Hydroxy-citronellal[1],[2]	Conflicting info.	Aldehyde
638011	Citral[1],[2]	Conflicting info.	Aldehyde
5283349	E,E-2,4-decadienal[1],[3]	Conflicting info.	Aldehyde
10947	Muscone[1],[6]	Conflicting info.	Ketone
8129	Heptanol[1],[2]	Conflicting info.	Alcohol
6276	Pentanol[1],[2],[8]	Conflicting info.	Alcohol
264	Butyric acid[1],[3],[8]	Conflicting info.	Acid
5374527	Beta-damascone[1],[3], [8],[5]	Conflicting info.	Ketone
6549	Linalool[1],[3], [8],[5], [2]	Conflicting info.	Alcohol
27458	2,3-diethyl pyrazine [1],[5]	Conflicting info.	Pyrazine
64832	Bourgeonal [1],[9]	Conflicting info.	Aldehyde
26447	Menthone[1],[3], [2]	Conflicting info.	Ketone
8051	2-heptanone[8] ,[1], [2]	Conflicting info.	Ketone
8158	Nonanoic acid[8],[1],[2]	Conflicting info.	Acid
957	Octanol[8],[1],[2]	Conflicting info.	Alcohol
637566	Geraniol [1],[2],[3],[9]	Conflicting info.	Alcohol
5362620	Z-4-decenal[1],[3]	Conflicting info.	Aldehyde
61346	1-octen-3-one[1],[3]	Conflicting info.	Ketone
8175	Decanal[8],[1],[3],[2],[5]	Conflicting info.	Aldehyde
8130	Heptanal[1],[2]	Conflicting info.	Aldehyde
454	Octanal[1],[3],[2]	Conflicting info.	Aldehyde
1032	Propionic acid[8],[3],[2]	Conflicting info.	Acid
10886	Amyl hexanoate[8],[2]	Conflicting info.	Ester
241	Benzene[8],[5],[2]	Conflicting info.	Cyclic compound
91604	Lyral [8],[5],[2]	Conflicting info.	Aldehyde
10890	Amyl butyrate[2],[7]	Conflicting info.	Ester
31289	Nonanal[8],[3],[2],[1]	Conflicting info.	Aldehyde
13187	2-nonanone[3],[5],[2]	Conflicting info.	Ketone
8144	Octanethiol[8],[5],[2]	Conflicting info.	Sulphur compound
11614	Butyl formate[8],[5],[2]	Conflicting info.	Ester

Pubchem_CID	Odorant name	Class	Chemical nature
8461	2,4-dinitrotoluene[8],[5]	Conflicting info.	Cyclic compound
106997	2-ethylfenchol[8],[5]	Conflicting info.	Alcohol
6852393	Androstenone[8],[5]	Conflicting info.	Ketone
5281167	Cis-3-hexen-1-ol[8],[3],[5]	Conflicting info.	Alcohol
20499	Coffee difuran[8],[5]	Conflicting info.	Cyclic compound
61014	Ethylene brassylate[5],[6]	Conflicting info.	Ester
3314	Eugenol[8],[9],[3],[5]	Conflicting info.	Cyclic compound
7127	Eugenol methyl ether[8],[3],[5],[7]	Conflicting info.	Cyclic compound
8467	Ethyl vanillin[8],[3],[5]	Conflicting info.	Cyclic compound
1549026	Geranyl acetate[8],[5]	Conflicting info.	Ester
6558	Isobutyl amine[8],[5]	Conflicting info.	Alkane
5460291	Laevo-arabinose[8],[5]	Conflicting info.	Aldehyde
62131	Methyl furfuryl disulfide[8],[5]	Conflicting info.	Sulphur compound
61294	Octyl octanoate[8],[5]	Conflicting info.	Ester
9261	Pyrazine[8],[5]	Conflicting info.	Pyrazine
17100	Terpineol[3],[5]	Conflicting info.	Alcohol
111037	Terpinyl acetate[8],[5]	Conflicting info.	Ester
527	Propanal[8],[3]	Conflicting info.	Aldehyde
12232	Dimethyl disulfide[8],[3]	Conflicting info.	Sulphur compound
6989	Thymol[9],[7]	Conflicting info.	Cyclic compound
4133	Methyl salicylate[8],[9]	Conflicting info.	Ester
5367530	E,E-2,4- dodecadienal[1]	Non-agonist	Aldehyde
1551246	Citralva[1]	Non-agonist	Nitrile compound
7410	Acetophenone[1]	Non-agonist	Ketone
196	Adipic acid[1]	Non-agonist	Acid
1110	Succinic acid[1]	Non-agonist	Acid
21648	Raspberry ketone[1]	Non-agonist	Ketone
8012	Butane thiol[1]	Non-agonist	Sulphur compound
8103	Hexanol[1]	Non-agonist	Alcohol
552003	2-bromo octanoic acid[1]	Non-agonist	Acid
8094	Heptanoic acid[1]	Non-agonist	Acid
12013	2-bromohexanoic acid[1]	Non-agonist	Acid
6184	Hexanal[1]	Non-agonist	Aldehyde
31229	Phenyl acetate[1]	Non-agonist	Ester
10106	1,4- cineole[1]	Non-agonist	Alkane
8892	Hexanoic acid[1]	Non-agonist	Acid
31245	Methyl pentanal[1]	Non-agonist	Aldehyde
11446	2-bromovaleric acid[1]	Non-agonist	Acid
10402	Citronellic acid[1]	Non-agonist	Acid
7519	Anisole[1]	Non-agonist	Cyclic compound
31276	Isoamyl acetate[1]	Non-agonist	Ester

**Pubchem_CID**	Odorant name	**Class**	**Chemical nature**
10430	Isovaleric acid[1]	Non-agonist	Acid
2758	1,8- cineole[1]	Non-agonist	Alkane
31268	Pyrrolidine[1]	Non-agonist	Cyclic compound
6655	Bromobutyric acid[1]	Non-agonist	Acid
7991	Valeric acid[1]	Non-agonist	Acid
8314	2-methylbutyric acid[1]	Non-agonist	Acid
5366074	Damascenone[1]	Non-agonist	Ketone
643731	E,Z-2,6- nonadienal[1]	Non-agonist	Aldehyde
5282108	A-ionone[1]	Non-agonist	Ketone
636687	E,E-26-nonadienal[1]	Non-agonist	Aldehyde
1201529	(R)-(-)-myrtenal[1]	Non-agonist	Aldehyde
638014	B-ionone[1]	Non-agonist	Ketone
1549025	Nerylacetate[1]	Non-agonist	Ester
5283339	E,E-2,4-nonadienal[1]	Non-agonist	Aldehyde
5281516	Farnesene[1]	Non-agonist	Terpene
32594	2-isobutyl-3-methoxypyrazine[1]	Non-agonist	Pyrazine
33166	2-isopropyl-3-methoxypyrazine[1]	Non-agonist	Pyrazine
5283335	2-enonenal[1]	Non-agonist	Aldehyde
15394	Ethyl vinyl ketone[1], [3]	Non-agonist	Ketone
28905	2,3-dietyl-5-methylpyrazine[1]	Non-agonist	Pyrazine
19309	Furaneol[1]	Non-agonist	Ketone
18635	Methional[1]	Non-agonist	Aldehyde
6561	Isobutylraldehyde[1]	Non-agonist	Aldehyde
1146	Trimethylamine[1]	Non-agonist	Methylamine
7976	2-methylpyrazine[1]	Non-agonist	Pyrazine
26331	2-ethylpyrazine[1]	Non-agonist	Pyrazine
26808	Trimethyl pyrazine[1]	Non-agonist	Pyrazine
520098	3-secbutyl-2-methoxypyrazine[1]	Non-agonist	Pyrazine
7363	Furfurylthiol[1]	Non-agonist	Sulphur compound
445070	Farnesol[1]	Non-agonist	Alcohol
22201	2,3-dimethylpyrazine[1]	Non-agonist	Pyrazine
8914	Nonanol[1]	Non-agonist	Alcohol
7335	(-)Carvyl acetate mix[1]	Non-agonist	Ester
379	Octanoic acid[1]	Non-agonist	Acid
18827	1-octen-3-ol[1]	Non-agonist	Alcohol
10448	Methionol[1]	Non-agonist	Alcohol
650	Diacetyl[1]	Non-agonist	Ketone
10900	1,2-dichlorethylene[1]	Non-agonist	Alkene
444294	(-)-Camphor[2]	Non-agonist	Ketone
785329	(+)-2-Phenylbutyric acid[2]	Non-agonist	Acid
159055	(+)-Camphor[2]	Non-agonist	Ketone

Pubchem_CID	Odorant name	Class	Chemical nature
785330	(-)-2-Phenylbutyric acid[2]	Non-agonist	Acid
82229	(-)-Fenchone[2]	Non-agonist	Ketone
1201521	(+)-Fenchone[2]	Non-agonist	Ketone
68382	2-coumaranone[2]	Non-agonist	Ketone
7895	2-pentanone[2]	Non-agonist	Ketone
54682930	4-hydroxycoumarin[2]	Non-agonist	Ketone
9309	Allyl benzene[2]	Non-agonist	Cyclic compound
7967	Cyclohexanone[2]	Non-agonist	Cyclic compound
24114	Butyl butyryl lactate[2]	Non-agonist	Ester
8468	Vanillic acid[2]	Non-agonist	Acid
12756	Γ-caprolactone[2]	Non-agonist	Ketone
2266	Nonanedioic acid[2]	Non-agonist	Acid
261	Butanal[2]	Non-agonist	Aldehyde
8063	Pentanal[2]	Non-agonist	Aldehyde
1031	1-propanol[2]	Non-agonist	Alcohol
2724897	(+)-2-heptanol[2]	Non-agonist	Alcohol
80080	(R)-(-)-2-octanol[2]	Non-agonist	Alcohol
2723888	(S)-(+)-2-octanol[2]	Non-agonist	Alcohol
8184	1-undecanol[2]	Non-agonist	Alcohol
12041	Ethyl pyruvate[2]	Non-agonist	Ester
61304	Heptyl isobutyrate[2]	Non-agonist	Ester
21596	Butyl heptanoate[2]	Non-agonist	Ester
7770	Propyl butyrate[2]	Non-agonist	Ester
88561	Tert-Butyl propionate[2]	Non-agonist	Ester
6569	2-butanone[2]	Non-agonist	Ketone
442495	(+)-Pulegone[2]	Non-agonist	Ketone
14505	2-Furyl methyl ketone[2]	Non-agonist	Ketone
31358	Dimedone[2]	Non-agonist	Ketone
263	1-butanol[2]	Non-agonist	Alcohol
7765	Acetal[2]	Non-agonist	Aldehyde
19707	2,3-hexanedione[2]	Non-agonist	Ketone
11583	2-hexanone[2]	Non-agonist	Ketone
62539	3,4-hexanedione[2]	Non-agonist	Ketone
8908	Hexyl acetate[2]	Non-agonist	Ester
1133	Thioglycolic acid[2]	Non-agonist	Acid
12348	N-amyl acetate[5]	Non-agonist	Ester
14489	Prenyl acetate[2]	Non-agonist	Ester
12180	Methyl butyrate[2]	Non-agonist	Ester
2969	Decanoic acid / capric acid[2]	Non-agonist	Acid
323	Coumarin[2]	Non-agonist	Cyclic compound
61809	2-ethoxythiazole[5]	Non-agonist	Sulphur compound

**Pubchem_CID**	Odorant name	**Class**	**Chemical nature**
18467	2-methoxypyrazine[5]	Non-agonist	Pyrazine
165675	(+)-Menthol[5]	Non-agonist	Alcohol
31244	Anisaldehyde[5]	Non-agonist	Cyclic compound
520119	1,3-butane dithiol[5]	Non-agonist	Sulphur compound
61431	1,1-dimethoxy-octane[5]	Non-agonist	Alkane
62835	Caramel furanone[5]	Non-agonist	Ketone
1068	Dimethyl sulfide[5]	Non-agonist	Sulphur compound
460	Guaiacol[5]	Non-agonist	Cyclic compound
10558	Isobutyl mercaptan[5]	Non-agonist	Sulphur compound
16255	Isoamyl octanoate[5]	Non-agonist	Ester
11124	Methyl propionate[5]	Non-agonist	Ester
5365991	Neryl isobutyrate[5]	Non-agonist	Ester
998	Phenyl acetaldehyde[5]	Non-agonist	Aldehyde
444212	Trans-Aconitic acid[5]	Non-agonist	Acid
181287	2,4,5-trimethyl-2,5-dihydro-1,3-thiazole[5]	Non-agonist	Cyclic compound
8186	Undecanal[5]	Non-agonist	Aldehyde
1183	Vanillin[5]	Non-agonist	Aldehyde
7704	(R/S)-γ-Octalactone[3]	Non-agonist	Ketone
10976	2-heptanol[3]	Non-agonist	Alcohol
8500	4-Methyl acetophenone[3]	Non-agonist	Ketone
5282110	Cinnamyl acetate[3]	Non-agonist	Ester
637520	Methyl cinnamate[3]	Non-agonist	Ester
62385	P,α-Dimethylstyrene[3]	Non-agonist	Cyclic compound
5283316	(E)-2-heptenal[3]	Non-agonist	Aldehyde
7342	Ethyl isobutyrate[3]	Non-agonist	Ester
11747	2,3-pentanedione[3]	Non-agonist	Ketone
5362588	Filbertone[3]	Non-agonist	Ketone
11086	2'-aminoacetophenone[3]	Non-agonist	Ketone
27470	3-Methylheptan-4-one[3]	Non-agonist	Ketone
179	Acetoin[3]	Non-agonist	Ketone
8369	Maltol[3]	Non-agonist	Ketone
10748	7-methoxycoumarin[3]	Non-agonist	Ketone
6736	3-Methyl-1H-indole[3]	Non-agonist	Cyclic compound
169110	2-Acetyl-2-thiazoline[3]	Non-agonist	Ketone
529251	2-propionylpyrroline[3]	Non-agonist	Ketone
33931	Homofuraneol[3]	Non-agonist	Ketone
61325	4-Methoxy-2,5-dimethyl-3(2H)-furanone[3]	Non-agonist	Ketone
62089	4-Methyloctanoic acid[3]	Non-agonist	Acid
61840	4-Ethyloctanoic acid[3]	Non-agonist	Acid
176	Acetic acid[3]	Non-agonist	Acid
6590	Isobutyric acid[3]	Non-agonist	Acid

**Pubchem_CID**	Odorant name	**Class**	**Chemical nature**
61199	5-Ethyl-3-hydroxy-4-methylfuran-2(5H)-one[3]	Non-agonist	Ketone
637542	P-Coumaric acid[3]	Non-agonist	Acid
999	2-Phenylacetic acid[3]	Non-agonist	Acid
107	3-Phenyl propionic acid[3]	Non-agonist	Acid
702	Ethanol[3]	Non-agonist	Alcohol
5315892	Cinnamyl alcohol[3]	Non-agonist	Alcohol
6054	2-phenylethanol[3]	Non-agonist	Alcohol
5362833	(2E,6Z)-Nona-2,6-dien-1-ol[3]	Non-agonist	Alcohol
31260	3-Methyl-1-butanol[3]	Non-agonist	Alcohol
8723	2-methyl-1-butanol[3]	Non-agonist	Alcohol
6560	Isobutyl alcohol[3]	Non-agonist	Alcohol
853433	Isoeugenol[3]	Non-agonist	Cyclic compound
326	Cuminaldehyde[3]	Non-agonist	Aldehyde
12777	6-Propyloxan-2-one[3]	Non-agonist	Ketone
12813	5-Hexyloxolan-2-one[3]	Non-agonist	Ketone
12810	Delta-decalactone[3]	Non-agonist	Ketone
7714	Gamma-undecalactone[3]	Non-agonist	Ketone
12844	Delta-dodecalactone[3]	Non-agonist	Ketone
16821	5-Octyloxolan-2-one[3]	Non-agonist	Ketone
62900	5-Butyl-4-methyloxolan-2-one[3]	Non-agonist	Ketone
31253	Myrcene[3]	Non-agonist	Terpene
7460	Alpha-phellandrene[3]	Non-agonist	Terpene
443162	(-)-alpha-Terpineol[3]	Non-agonist	Alcohol
440968	(-)-alpha-Pinene[3]	Non-agonist	Terpene
82227	(+)-alpha-Pinene[3]	Non-agonist	Terpene
440967	(-)-beta-Pinene[3]	Non-agonist	Terpene
10290825	(+)-beta-Pinene[3]	Non-agonist	Terpene
10887971	(+)-Sabinene[3]	Non-agonist	Terpene
27866	Rose oxide[3]	Non-agonist	Cyclic compound
12101	3-ethylphenol[3]	Non-agonist	Cyclic compound
31242	4-ethylphenol[3]	Non-agonist	Cyclic compound
335	2-methylphenol[3]	Non-agonist	Cyclic compound
342	3-methylphenol[3]	Non-agonist	Cyclic compound
2879	4-methylphenol[3]	Non-agonist	Cyclic compound
7771	2,4-dimethylphenol[3]	Non-agonist	Cyclic compound
11335	2,6-dimethylphenol[3]	Non-agonist	Cyclic compound
62453	4-ethenylphenol[3]	Non-agonist	Cyclic compound
332	4-vinylguaiacol[3]	Non-agonist	Cyclic compound
7041	2,6-dimethoxyphenol[3]	Non-agonist	Cyclic compound
7144	2-methoxy-4-methylphenol[3]	Non-agonist	Cyclic compound
14519	2-Methoxy-5-methylphenol[3]	Non-agonist	Cyclic compound

**Pubchem_CID**	Odorant name	**Class**	**Chemical nature**
62465	2-Methoxy-4-ethylphenol[3]	Non-agonist	Cyclic compound
17739	2-methoxy-4-propylphenol[3]	Non-agonist	Cyclic compound
30914	2-acetylpyrazine[3]	Non-agonist	Pyrazine
6430888	2-Methyl-3-sulfanylpentan-1-ol[3]	Non-agonist	Alcohol
62237	3-Mercapto-2-pentanone[3]	Non-agonist	Ketone
529635	2-Sulfanylpentan-3-one[3]	Non-agonist	Ketone
61592	Ethyl 3-methylthiopropionate[3]	Non-agonist	Ester
34286	2-Methyl-3-furanthiol[3]	Non-agonist	Sulphur compound
47649	Methyl 2-methyl-3-furyl disulfide[3]	Non-agonist	Sulphur compound
15380	Bis(methylthio)methane[3]	Non-agonist	Sulphur compound
80408	Thenyl mercaptan[3]	Non-agonist	Sulphur compound
6427135	1-p-Menthene-8-thiol[3]	Non-agonist	Sulphur compound
521348	3-Sulfanylhexan-1-ol[3]	Non-agonist	Alcohol
526487	3-Mercapto-3-methylbutyl formate[3]	Non-agonist	Ester
88290	4-Mercapto-4-methyl-2-pentanone[3]	Non-agonist	Ketone
526195	4-Methoxy-2-methyl-2-butanethiol[3]	Non-agonist	Sulphur compound
19310	Dimethyl trisulfide[3]	Non-agonist	Sulphur compound
5319765	Methyl propyl trisulfide[3]	Non-agonist	Sulphur compound
12377	Propyl disulfide[3]	Non-agonist	Sulphur compound
22383	Dipropyl trisulfide[3]	Non-agonist	Sulphur compound
66282	Allyl methyl sulfide[3]	Non-agonist	Sulphur compound
16590	Allyl disulfide[3]	Non-agonist	Sulphur compound
7284	2-methylbutyraldehyde[3]	Non-agonist	Aldehyde
11552	Isovaleraldehyde[3]	Non-agonist	Aldehyde
5281168	2-hexenal, (2e)- [3]	Non-agonist	Aldehyde
643941	3-hexenal, (3z)- [3]	Non-agonist	Aldehyde
5362814	4-heptenal, (4z)- [3]	Non-agonist	Aldehyde
6427080	2-octenal, (2z)- [3]	Non-agonist	Aldehyde
5354833	2-nonenal, (2z)- [3]	Non-agonist	Aldehyde
6429282	2,4-nonadienal, (2e,4z)- [3]	Non-agonist	Aldehyde
5283345	2-decenal, (2e)- [3]	Non-agonist	Aldehyde
3018619	12-methyltridecanal[3]	Non-agonist	Aldehyde
5283356	2-undecenal[3]	Non-agonist	Aldehyde
7799	Ethyl octanoate[3]	Non-agonist	Ester
8048	Ethyl decanoate[3]	Non-agonist	Ester
7165	Ethyl benzoate[3]	Non-agonist	Ester
177	Acetaldehyde[3]	Non-agonist	Aldehyde
7150	Methyl benzoate[3]	Non-agonist	Ester
7762	Ethyl butyrate[3]	Non-agonist	Ester
10882	Ethyl pentanoate[3]	Non-agonist	Ester
637758	Ethyl cinnamate[3]	Non-agonist	Ester

**Pubchem_CID**	Odorant name	**Class**	**Chemical nature**
24020	Ethyl 2-methylbutyrate[3]	Non-agonist	Ester
7945	Ethyl isovalerate[3]	Non-agonist	Ester
117477	Ethyl 4-methylvalerate[3]	Non-agonist	Ester
61293	Ethyl 3-hydroxyhexanoate[3]	Non-agonist	Ester
13357	Methyl 2-methylbutyrate[3]	Non-agonist	Ester
11039	Methyl isobutyrate[3]	Non-agonist	Ester
12209	2-Methylbutyl acetate[3]	Non-agonist	Ester
8857	Ethyl acetate[3]	Non-agonist	Ester
31272	Butyl acetate [3]	Non-agonist	Ester
5363388	3-Hexenyl acetate, (3Z)-[3]	Non-agonist	Ester
10409	Cyclopentadecanone[6]	Non-agonist	Ketone
5362739	Globanone[6]	Non-agonist	Ketone
5317881	Ambrettolide[6]	Non-agonist	Ketone
235414	Exaltolide[6]	Non-agonist	Ketone
11107424	Habanolide[6]	Non-agonist	Ketone
6669	Musk ketone[6]	Non-agonist	Ketone
91497	Galaxolide[6]	Non-agonist	Cyclic compound
89440	Tonalide[6]	Non-agonist	Ketone
92292	Cashmeran[6]	Non-agonist	Ketone
16063567	Helvetolide[6]	Non-agonist	Ester
67525	Cyclopentadecane[6]	Non-agonist	Cyclic compound
107327	Cyclopentadecanol[6]	Non-agonist	Alcohol
73918	Cyclodecanone[6]	Non-agonist	Ketone
13420	Cycloundecanone[6]	Non-agonist	Ketone
61303	2-pentadecanone[6]	Non-agonist	Ketone
13162	8-pentadecanone[6]	Non-agonist	Ketone
996	Phenol[9]	Non-agonist	Cyclic compound
7136	Eugenyl acetate[8]	Non-agonist	Ester
8635	Methyl anthranilate[7]	Non-agonist	Ester
19835	1,9 nonanediol[7]	Non-agonist	Alcohol
31252	2,5 dimethyl pyrazine[7]	Non-agonist	Pyrazine
5281517	Trans-beta-farnesene[7]	Non-agonist	Terpene
62375	Benzaldehyde dimethyl acetal[7]	Non-agonist	Aldehyde
798	Indole[7]	Non-agonist	Cyclic compound
7731	4-methylanisole[7]	Non-agonist	Cyclic compound
229888	Phenethyl octanoate[7]	Non-agonist	Ester
8091	Methyl octanoate[7]	Non-agonist	Ester

Table S2: Experimentally tested odorants for OR2W1

Pubchem_CID	Odorant name	Class	Chemical nature
8106	Hexane-1- thiol[10]	Agonist	Sulphur compound
15422	Heptane-1- thiol [10]	Agonist	Sulphur compound
8067	Pentane-1- thiol [10]	Agonist	Sulphur compound
439570	(-)-Carvone[2], [3]	Agonist	Ketone
16724	(+)-Carvone[5],[8]	Agonist	Ketone
22227	(+)-Dihydrocarvone[2]	Agonist	Ketone
440917	(R)-(+)-limonene[3],[5], [8]	Agonist	Terpene
6992244	(R)-1-octen-3-ol ((R/S)-1-Octen-3-ol)[3]	Agonist	Alcohol
7704	(R/S)-γ-Octalactone[3]	Agonist	Ketone
7793	(S)-(-)-citronellol[2]	Agonist	Alcohol
439250	(S)-(−)-limonene[3]	Agonist	Terpene
8174	1-decanol[2]	Agonist	Alcohol
8129	1-heptanol[2]	Agonist	Alcohol
8914	1-nonanol[2], [3]	Agonist	Alcohol
957	1-octanol[2], [8]	Agonist	Alcohol
19707	2,3-hexanedione[2]	Agonist	Ketone
10976	2-heptanol[3]	Agonist	Alcohol
11583	2-hexanone[2]	Agonist	Ketone
13187	2-nonanone[3], [2]	Agonist	Ketone
8093	2-octanone[2]	Agonist	Ketone
16800	2-pentylpyridine[3]	Agonist	Cyclic compound
78126	2-phenylethane thiol [3]	Agonist	Sulphur compound
7654	2-Phenylethyl acetate[3]	Agonist	Ester
62539	3,4-hexanedione[2]	Agonist	Ketone
7802	3-heptanone[2]	Agonist	Ketone
518810	3-Mercaptohexyl acetate[3]	Agonist	Ester
31276	3-Methylbutyl acetate/ isoamyl acetate[3]	Agonist	Ester
246728	3-octanone[2]	Agonist	Ketone
68110	4-chromanone[2]	Agonist	Ketone
8500	4-Methyl acetophenone[3]	Agonist	Ketone
76354	6-Methyl-2,4-heptanedione[3]	Agonist	Ketone
7410	Acetophenone[2]	Agonist	Ketone
15717	Allyl phenylacetate[2], [3],[5],[8]	Agonist	Ester
3102	Benzophenone[2]	Agonist	Cyclic compound
8785	Benzyl acetate[2],[11]	Agonist	Ester
5282110	Cinnamyl acetate[3]	Agonist	Ester
62378	Dihydrojasmone[2]	Agonist	Ketone
8815	Estragole[3]	Agonist	Cyclic compound
18686	Ethyl cyclohexanecarboxylate[3]	Agonist	Ester
8130	Heptanal[2]	Agonist	Aldehyde
637520	Methyl cinnamate[3]	Agonist	Ester
8908	Hexyl acetate[2], [3]	Agonist	Ester
31289	Decanoic acid[2], [3],[8]	Agonist	Acid

**Pubchem_CID**	Odorant name	**Class**	**Chemical nature**
15077	Nonane thiol[2]	Agonist	Sulphur compound
8158	Nonanoic acid[2], [8]	Agonist	Acid
454	Octanal[2], [3]	Agonist	Aldehyde
8144	Octane thiol[2] , [5], [8]	Agonist	Sulphur compound
62385	P,α-Dimethylstyrene[3]	Agonist	Cyclic compound
14489	Prenyl acetate[2]	Agonist	Ester
637563	Trans-anethole[3]	Agonist	Cyclic compound
64805	Helional[8]	Agonist	Aldehyde
20499	Coffee difuran[5] ,[8]	Agonist	Cyclic compound
7136	Eugenyl acetate[8]	Agonist	Ester
4133	Methyl salicylate[8]	Agonist	Ester
5283316	(E)-2-heptenal[3]	Agonist	Aldehyde
61431	1,1-dimethoxy-octane[5]	Agonist	Alkane
80314	2,4-nonanedione[3]	Agonist	Ketone
84192	2,4-octanedione[3]	Agonist	Ketone
61809	2-ethoxythiazole[5]	Agonist	Thiazole
61052	3-phenyl propyl propionate[5]	Agonist	Ester
8635	Methyl anthranilate[7]	Agonist	Ester
24433	Butyl anthranilate[5]	Agonist	Ester
7794	Citronellal[11]	Agonist	Aldehyde
12348	N- amyl acetate[5]	Agonist	Ester
1549026	Geranyl acetate[5] ,[8]	Conflicting info.	Ester
8103	1-hexanol[2] ,[3]	Conflicting info.	Alcohol
165675	(+)-Menthol[8],[5]	Conflicting info.	Alcohol
323	Coumarin[2],[3], [5, 11]	Conflicting info.	Ketone
379	Octanoic acid[2] ,[3]	Conflicting info.	Acid
2969	Capric acid[2] ,[3]	Conflicting info.	Acid
6184	Hexanal[2] ,[3]	Conflicting info.	Aldehyde
106997	2-ethyl fenchol [5] ,[8]	Conflicting info.	Alcohol
637566	Geraniol[2] ,[3]	Conflicting info.	Alcohol
8467	Ethyl vanillin[3], [5],[8]	Conflicting info.	Aldehyde
1183	Vanillin[3], [5]	Conflicting info.	Aldehyde
6276	1-pentanol[2], [8]	Conflicting info.	Alcohol
264	Butyric acid[8]	Conflicting info.	Acid
3314	Eugenol[3], [5],[8]	Conflicting info.	Cyclic compounds
8051	2-heptanone[2], [8]	Conflicting info.	Ketone
5281167	Cis-3-hexenol[3], [5],[8], [12]	Conflicting info.	Alcohol
111037	Terpinyl acetate[8], [5]	Conflicting info.	Ester
638011	Citral[8], [11]	Conflicting info.	Aldehyde
71309167	Propanal[3], [8]	Conflicting info.	Aldehyde
11614	Butyl formate[2], [8]	Conflicting info.	Ester
17100	Alpha-terpineol[3], [5]	Conflicting info.	Alcohol
785330	(-)-2-Phenylbutyric acid[2]	Non-agonist	Acid
444294	(-)-Camphor[2]	Non-agonist	Ketone

**Pubchem_CID**	Odorant name	**Class**	**Chemical nature**
62131	Methyl furfuryl disulphide[8]	Non-agonist	Cyclic compound
82229	(-)-Fenchone[2], [3]	Non-agonist	Ketone
785329	(+)-2-Phenylbutyric acid[2]	Non-agonist	Acid
159055	(+)-Camphor[2]	Non-agonist	Ketone
637511	Cinnamaldehyde[5]	Non-agonist	Aldehyde
1201521	(+)-Fenchone[2]	Non-agonist	Ketone
68382	2-coumaranone[2]	Non-agonist	Ketone
7895	2-pentanone[2]	Non-agonist	Ketone
529481	3-Methyl-2,4-nonanedione[3]	Non-agonist	Ketone
54682930	4-hydroxycoumarin[2]	Non-agonist	Ketone
9309	Allyl benzene[2]	Non-agonist	Cyclic compound
8878	Allyl heptanoate[2]	Non-agonist	Ester
10886	Amyl hexanoate [2], [8]	Non-agonist	Ester
241	Benzene[2], [5] ,[8]	Non-agonist	Cyclic compound
7967	Cyclohexanone[2]	Non-agonist	Ketone
31265	Ethyl hexanoate[3]	Non-agonist	Ester
7342	Ethyl isobutyrate[2], [3]	Non-agonist	Ester
7590	Ethylphenyl acetate[3]	Non-agonist	Ester
24114	Butyl butyryl lactate[2]	Non-agonist	Ester
8094	Heptanoic acid[2]	Non-agonist	Acid
8892	Hexanoic acid[2], [3]	Non-agonist	Acid
91604	Lyral[2], [5] ,[8]	Non-agonist	Aldehyde
7991	Pentanoic acid[2], [3]	Non-agonist	Acid
31229	Phenyl acetate[2]	Non-agonist	Ester
1032	Propionic acid[2], [3],[8]	Non-agonist	Acid
8468	Vanillic acid[2]	Non-agonist	Acid
8175	Decanal[2], [3], [5],[8]	Non-agonist	Aldehyde
520119	1,3-butane di thiol [5]	Non-agonist	Sulphur compound
8461	2,4-DNT[5] ,[8]	Non-agonist	Cyclic compound
12756	Gamma-Caprolactone[2]	Non-agonist	Ketone
1133	Thioglycolic acid[5]	Non-agonist	Acid
2266	Nonanedioic acid[2]	Non-agonist	Acid
261	Butanal[2]	Non-agonist	Aldehyde
8063	Pentanal[2]	Non-agonist	Aldehyde
7765	Acetal[3]	Non-agonist	Aldehyde
7888	Hydroxycitronellal[2]	Non-agonist	Aldehyde
263	1-butanol[3]	Non-agonist	Alcohol
1031	1-propanol[2]	Non-agonist	Alcohol
2724897	(+)-2-heptanol[2]	Non-agonist	Alcohol
80080	(R)-(-)-2-octanol[2]	Non-agonist	Alcohol
2723888	(S)-(+)-2-octanol[2]	Non-agonist	Alcohol
6549	Linalool[3], [5], [8]	Non-agonist	Terpene
8184	1-undecanol[2]	Non-agonist	Alcohol
12041	Ethyl pyruvate[2]	Non-agonist	Ester

**Pubchem_CID**	Odorant name	**Class**	**Chemical nature**
61304	Heptyl isobutyrate[2]	Non-agonist	Ester
21596	Butyl heptanoate[2]	Non-agonist	Ester
10890	Amyl butyrate[2]	Non-agonist	Ester
7770	Propyl butyrate[2]	Non-agonist	Ester
12180	Methyl butyrate[3]	Non-agonist	Ester
88561	Tert-Butyl propionate[2]	Non-agonist	Ester
6569	2-butanone[2]	Non-agonist	Ketone
442495	(+)-Pulegone[2]	Non-agonist	Ketone
14505	2-Furyl methyl ketone[2]	Non-agonist	Ketone
31358	Dimedone[2]	Non-agonist	Ketone
26447	(-)-Menthone[2]	Non-agonist	Ketone
650	2,3-butanedione[3]	Non-agonist	Ketone
11747	2,3-pentanedione[3]	Non-agonist	Ketone
5362588	Filbertone[3]	Non-agonist	Ketone
11086	2'-aminoacetophenone[3]	Non-agonist	Ketone
61346	1-octen-3-one[3]	Non-agonist	Ketone
15394	Ethyl vinyl ketone[3]	Non-agonist	Ketone
27470	3-Methylheptan-4-one[3]	Non-agonist	Ketone
179	Acetoin[3]	Non-agonist	Ketone
8369	Maltol[3]	Non-agonist	Alcohol
10748	7-methoxycoumarin[3]	Non-agonist	Ketone
6736	3-Methyl-1H-indole[3]	Non-agonist	Cyclic compound
169110	2-Acetyl-2-thiazoline[3]	Non-agonist	Thiazole
529251	2-propionylpyrroline[3]	Non-agonist	Cyclic compound
19309	Furaneol[3]	Non-agonist	Cyclic compound
33931	Homofuraneol[3]	Non-agonist	Cyclic compound
61325	4-Methoxy-2,5-dimethyl-3(2H)-furanone[3]	Non-agonist	Cyclic compound
62835	Sotolon[3], [5]	Non-agonist	Cyclic compound
61199	5-Ethyl-3-hydroxy-4-methylfuran-2(5H)-one[3]	Non-agonist	Ketone
6590	Isobutyric acid[3]	Non-agonist	Acid
8314	2-Methylbutanoic acid[3]	Non-agonist	Acid
62089	4-Methyloctanoic acid[3]	Non-agonist	Acid
61840	4-Ethyloctanoic acid[3]	Non-agonist	Acid
176	Acetic acid[3]	Non-agonist	Acid
637542	P-Coumaric acid[3]	Non-agonist	Acid
999	2-Phenylacetic acid[3]	Non-agonist	Acid
107	3-Phenyl propionic acid[3]	Non-agonist	Acid
702	Ethanol[3]	Non-agonist	Alcohol
5315892	Cinnamyl alcohol[3]	Non-agonist	Alcohol
6054	2-phenylethanol[3]	Non-agonist	Alcohol
5362833	(2E,6Z)-Nona-2,6-dien-1-ol[3]	Non-agonist	Alcohol
31260	3-Methyl-1-butanol[3]	Non-agonist	Alcohol
8723	2-methyl-1-butanol[3]	Non-agonist	Alcohol
6560	Isobutyl alcohol[3]	Non-agonist	Alcohol

Pubchem_CID	Odorant name	Class	Chemical nature
853433	Isoeugenol[3]	Non-agonist	Cyclic compound
7127	Methyl eugenol[3], [5] ,[8]	Non-agonist	Cyclic compound
31244	P-anisaldehyde[3], [5]	Non-agonist	Aldehyde
326	Cuminaldehyde[3]	Non-agonist	Aldehyde
12777	6-Propyloxan-2-one[3]	Non-agonist	Ketone
7710	5-Pentyloxolan-2-one[3]	Non-agonist	Ketone
12813	5-Hexyloxolan-2-one[3]	Non-agonist	Ketone
12810	Delta-decalactone[3]	Non-agonist	Ketone
7714	Gamma-undecalactone[3]	Non-agonist	Ketone
12844	Delta-Dodecalactone[3]	Non-agonist	Ketone
16821	5-Octyloxolan-2-one[3]	Non-agonist	Ketone
62900	5-Butyl-4-methyloxolan-2-one[3]	Non-agonist	Ketone
31253	Myrcene[3]	Non-agonist	Terpene
2758	1,8-cineol[3]	Non-agonist	Alcohol
7460	Alpha-Phellandrene[3]	Non-agonist	Terpene
443162	(-)-alpha-Terpineol[3]	Non-agonist	Alcohol
440968	(-)-alpha-Pinene[3]	Non-agonist	Terpene
82227	(+)-alpha-Pinene[3]	Non-agonist	Terpene
440967	(-)-beta-Pinene[3]	Non-agonist	Terpene
10290825	(+)-beta-Pinene[3]	Non-agonist	Terpene
10887971	(+)-sabinene[3]	Non-agonist	Terpene
5374527	(E)-beta-damascone[3], [8]	Non-agonist	Ketone
5366074	Beta-damascenone[3]	Non-agonist	Ketone
638014	Beta-Ionone[3]	Non-agonist	Ketone
27866	Rose oxide[3]	Non-agonist	Terpene
12101	3-ethylphenol[3]	Non-agonist	Cyclic compound
31242	4-ethylphenol[3]	Non-agonist	Cyclic compound
335	2-methylphenol[3]	Non-agonist	Cyclic compound
342	3-methylphenol[3]	Non-agonist	Cyclic compound
2879	4-methylphenol[3]	Non-agonist	Cyclic compound
7771	2,4-dimethylphenol[3]	Non-agonist	Cyclic compound
11335	2,6-dimethylphenol[3]	Non-agonist	Cyclic compound
460	Guaiacol[3], [5]	Non-agonist	Alcohol
7144	2-methoxy-4-methylphenol[3]	Non-agonist	Cyclic compound
14519	2-Methoxy-5-methylphenol[3]	Non-agonist	Cyclic compound
62465	2-Methoxy-4-ethylphenol[3]	Non-agonist	Cyclic compound
17739	2-methoxy-4-propylphenol[3]	Non-agonist	Cyclic compound
62453	4-ethenylphenol[3]	Non-agonist	Alcohol
332	4-vinylguaiacol[3]	Non-agonist	Alcohol
7041	2,6-dimethoxyphenol[3]	Non-agonist	Cyclic compound
28905	2,3-Diethyl-5-methylpyrazine[3]	Non-agonist	Pyrazine
26808	2,3,5-trimethylpyrazine[3]	Non-agonist	Pyrazine
62237	3-Mercapto-2-pentanone[3]	Non-agonist	Ketone
529635	2-Sulfanylpentan-3-one[3]	Non-agonist	Ketone

**Pubchem_CID**	Odorant name	**Class**	**Chemical nature**
32594	2-Isobutyl-3-methoxypyrazine[3]	Non-agonist	Pyrazine
33166	2-Isopropyl-3-methoxypyrazine[3]	Non-agonist	Pyrazine
520098	2-Sec-Butyl-3-methoxypyrazine[3]	Non-agonist	Pyrazine
30914	2-acetylpyrazine[3]	Non-agonist	Pyrazine
61592	Ethyl 3-methylthiopropionate[3]	Non-agonist	Ester
7363	Furfuryl mercaptan[3]	Non-agonist	Sulphur compound
34286	2-Methyl-3-furan thiol [3]	Non-agonist	Sulphur compound
6430888	2-Methyl-3-sulfanylpentan-1-ol[3]	Non-agonist	Alcohol
47649	Methyl 2-methyl-3-furyl disulfide[3]	Non-agonist	Sulphur compound
18635	3-methylthiopropionaldehyde[3]	Non-agonist	Aldehyde
10448	3-(Methylthio)-1-propanol[3]	Non-agonist	Alcohol
15380	Bis(methylthio)methane[3]	Non-agonist	Alkane
80408	Thenyl mercaptan[3]	Non-agonist	Sulphur compound
6427135	1-p-Menthene-8- thiol [3]	Non-agonist	Sulphur compound
521348	3-Sulfanylhexan-1-ol[3]	Non-agonist	Alcohol
526487	3-Mercapto-3-methylbutyl formate[3]	Non-agonist	Ester
88290	4-Mercapto-4-methyl-2-pentanone[3]	Non-agonist	Ketone
526195	4-Methoxy-2-methyl-2-butane thiol [3]	Non-agonist	Sulphur compound
1068	Methyl sulfide[3]	Non-agonist	Sulphur compound
12232	Dimethyl disulfide[3], [8]	Non-agonist	Sulphur compound
19310	Dimethyl trisulfide[3]	Non-agonist	Sulphur compound
5319765	Methyl propyl trisulfide[3]	Non-agonist	Sulphur compound
12377	Propyl disulfide[3]	Non-agonist	Sulphur compound
22383	Dipropyl trisulfide[3]	Non-agonist	Sulphur compound
66282	Allyl methyl sulfide[3]	Non-agonist	Sulphur compound
16590	Allyl disulfide[3]	Non-agonist	Sulphur compound
6561	Isobutyraldehyde[3]	Non-agonist	Aldehyde
7284	2-methylbutyraldehyde[3]	Non-agonist	Aldehyde
11552	Isovaleraldehyde[3]	Non-agonist	Aldehyde
5281168	2-hexenal, (2e)- [3]	Non-agonist	Aldehyde
643941	3-hexenal, (3z)- [3]	Non-agonist	Aldehyde
5362814	4-heptenal, (4z)- [3]	Non-agonist	Aldehyde
6427080	2-octenal, (2z)- [3]	Non-agonist	Aldehyde
5283335	2-nonenal, (2e)- [3]	Non-agonist	Aldehyde
5354833	2-nonenal, (2z)- [3]	Non-agonist	Aldehyde
5283339	2,4-nonadienal, (2e,4e)- [3]	Non-agonist	Aldehyde
6429282	2,4-nonadienal, (2e,4z)- [3]	Non-agonist	Aldehyde
643731	2,6-nonadienal, (2e,6z)- [3]	Non-agonist	Aldehyde
5283345	2-decenal, (2e)- [3]	Non-agonist	Aldehyde
5362620	4-decenal, (4z)- [3]	Non-agonist	Aldehyde
5283349	2,4-decadienal, (2e,4e)- [3]	Non-agonist	Aldehyde
3018619	12-methyltridecanal[3]	Non-agonist	Aldehyde
5283356	2-undecenal[3]	Non-agonist	Aldehyde
177	Acetaldehyde[3]	Non-agonist	Aldehyde

**Pubchem_CID**	Odorant name	**Class**	**Chemical nature**
998	Phenylacetaldehyde[3], [5]	Non-agonist	Aldehyde
7150	Methyl benzoate[3]	Non-agonist	Ester
7762	Ethyl butyrate[3]	Non-agonist	Ester
10882	Ethyl pentanoate[3]	Non-agonist	Ester
7799	Ethyl octanoate[3]	Non-agonist	Ester
8048	Ethyl decanoate[3]	Non-agonist	Ester
7165	Ethyl benzoate[3]	Non-agonist	Ester
637758	Ethyl cinnamate[3]	Non-agonist	Ester
24020	Ethyl 2-methylbutyrate[3]	Non-agonist	Ester
7945	Ethyl isovalerate[3]	Non-agonist	Ester
117477	Ethyl 4-methylvalerate[3]	Non-agonist	Ester
61293	Ethyl 3-hydroxyhexanoate[3]	Non-agonist	Ester
13357	Methyl 2-methylbutyrate[3]	Non-agonist	Ester
11039	Methyl isobutyrate[3]	Non-agonist	Ester
12209	2-Methylbutyl acetate[3]	Non-agonist	Ester
8857	Ethyl acetate[3]	Non-agonist	Ester
31272	Butyl acetate[3]	Non-agonist	Ester
5363388	3-Hexenyl acetate, (3Z)- [3]	Non-agonist	Ester
5460291	Arabinose, L-[8]	Non-agonist	Aldehyde
7047	Quinoline[5] ,[8]	Non-agonist	Cyclic compound
61294	Octyl octanoate[8]	Antagonist	Ester
6558	Isobutyl amine[8]	Antagonist	Alkane
6852393	Androstenone [5] ,[8]	Antagonist	Ketone
181287	2,4,5-trimethyl-3-thiazoline[5]	Antagonist	Thiazole
61014	Ethylene brassylate[5]	Antagonist	Ester
92979	Androstadienone[5]	Antagonist	Ketone
10430	Isovaleric acid[5]	Antagonist	Acid

```
               1.50                           2.50
1U19        MNGTEGPNFYVPFSNKTGVVRSPFEAPQYYLAEPWQFSMLAAYMFLLIML 50
OR1A1       ---MRENNQSSTLEFILLGVTGQ-----------QEQEDFFYILFLFIYPI 37
OR2W1       ----------MDQSNYSSLHGFILLGFSNHP---KMEMILSGVVAIFYLI 37
```

```
               1.50                           2.50
1U19        GFPINFLTLYVTVQHKKLRTPLNYILLNLAVADLFMVFGGFTTTLYTSLH 100
OR1A1       TLIGNLLIVLAICSDVRLHNPMYFLLANLSLVDIFFSSVTIPKMLANHLL 87
OR2W1       TLVGNTAIILASLLDSQLHTPMYFFLRNLSFLDLCFTTSIIPQMLVNLWG 87

                                              3.50
1U19        GYFVFGPTGCNLEGFFATLGGEIALWSLVVLAIERYVVVCKPMSNFR-FG 148
OR1A1       GSKSISFGGCLTQMYFMIALGNTDSYILAAMAYDRAVAISRPLHYTTIMS 137
OR2W1       PDKTISYVGCIIQLYVYMWLGSVECLLLAVMSYDRFTAICKPLHYFVVMN 137

                    4.50
1U19        ENHAIMGVAFTWVMALACAAPPLVGWSRYIPEGMQCS----CGIDYY--- 192
OR1A1       PRSCIWLIAGSWVIGNANALPHTLLTASLSFCGNQEVANFYCDITPLLKL 187
OR2W1       PHLCLKMIIMIWSISLANSVVLCTLTLNLPTCGNNILDHFLCELPALVKI 187

                               5.50
1U19        TPHEETNNESFVIYMFVVHFIIPLIVIFFCYGQLVFTVKEAAAQQQESAT 242
OR1A1       -SCSDIHFHVKMMYLGVGIFSVPLLCIIVSYIRVFSTVFQVPST------ 230
OR2W1       ACVDTTTVEMSVFALGIIIVLTPLILILISYGYIAKAVLRTKSK------ 231

                                 6.50
1U19        TQKAEKEVTRMVIIMVIAFLICWLPYAGVAFYIFTHQGSDFGPIFMTIPA 292
OR1A1       --KGVLKAFSTCGSHLTVVSLYYGTVMGTYFRPLTN----YSLKDAVITV 274
OR2W1       --ASQRKAMNTCGSHLTVVSMFYGTIIYMYLQPGNRA---SKDQGKFLTL 276

                   7.50
1U19        FFAKTSAVYNPVIYIMMNKQFRNCMVTTLCCGKNPLGDDEASTTVSKTET 342
OR1A1       MYTAVTPMLNPFIYSLRNRDMKAALRKLFNKRISS--------------- 309
OR2W1       FYTVITPSLNPLIYTLRNKDMKDALKKLMRFHHKSTKIKRNCKS------ 320

1U19        SQVAPA 348
OR1A1       ------
OR2W1       ------
```

Figure S1: Alignment of OR1A1, OR2W1 and 1U19 used for model building. GPCR conserved motifs are bold and central helix residues are shown in red colour while cysteine residues forming disulphide bond are in blue colour. Transmembrane regions are highlighted in yellow colour. Centre residues are numbered according to Ballesteros-Weinstein residue numbering scheme.

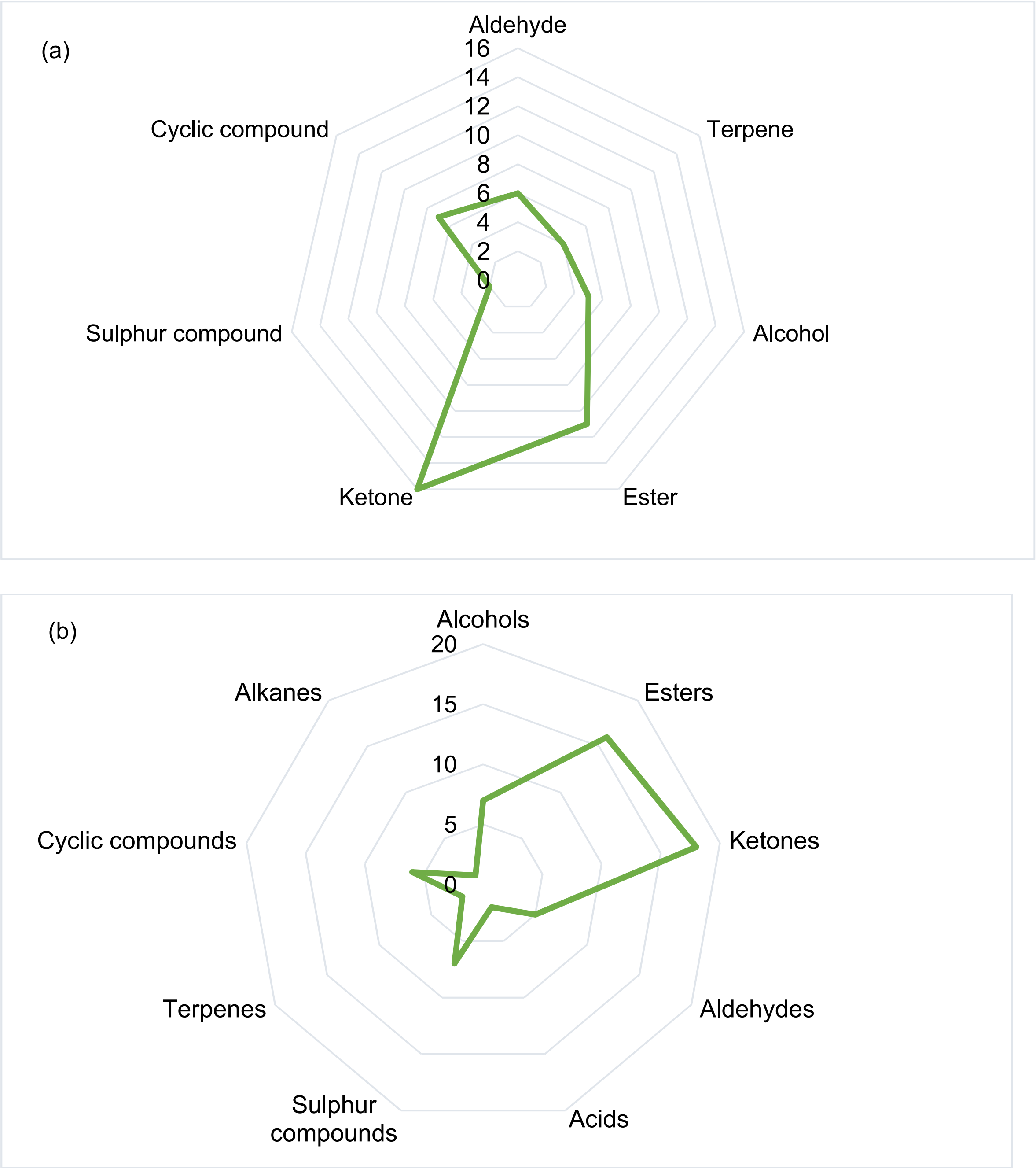

Figure S2: Diverse chemical nature of known agonists for (a) OR1A1 and (b) OR2W1

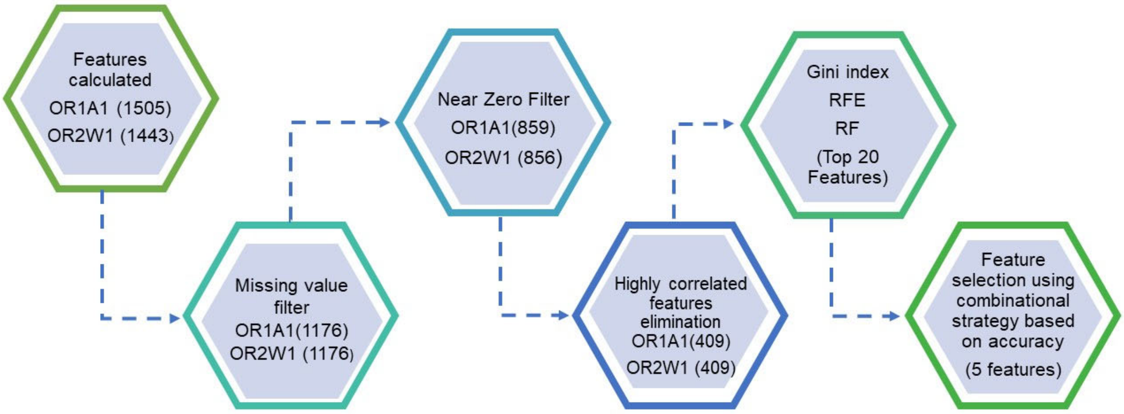

Figure S3: Workflow for feature selection for OR1A1 and OR2W1. Gini index is an example for filter feature selection method, RFE, recursive feature selection method is an example for the wrapper method, and RF, random forest is an example for embedded feature selection method.

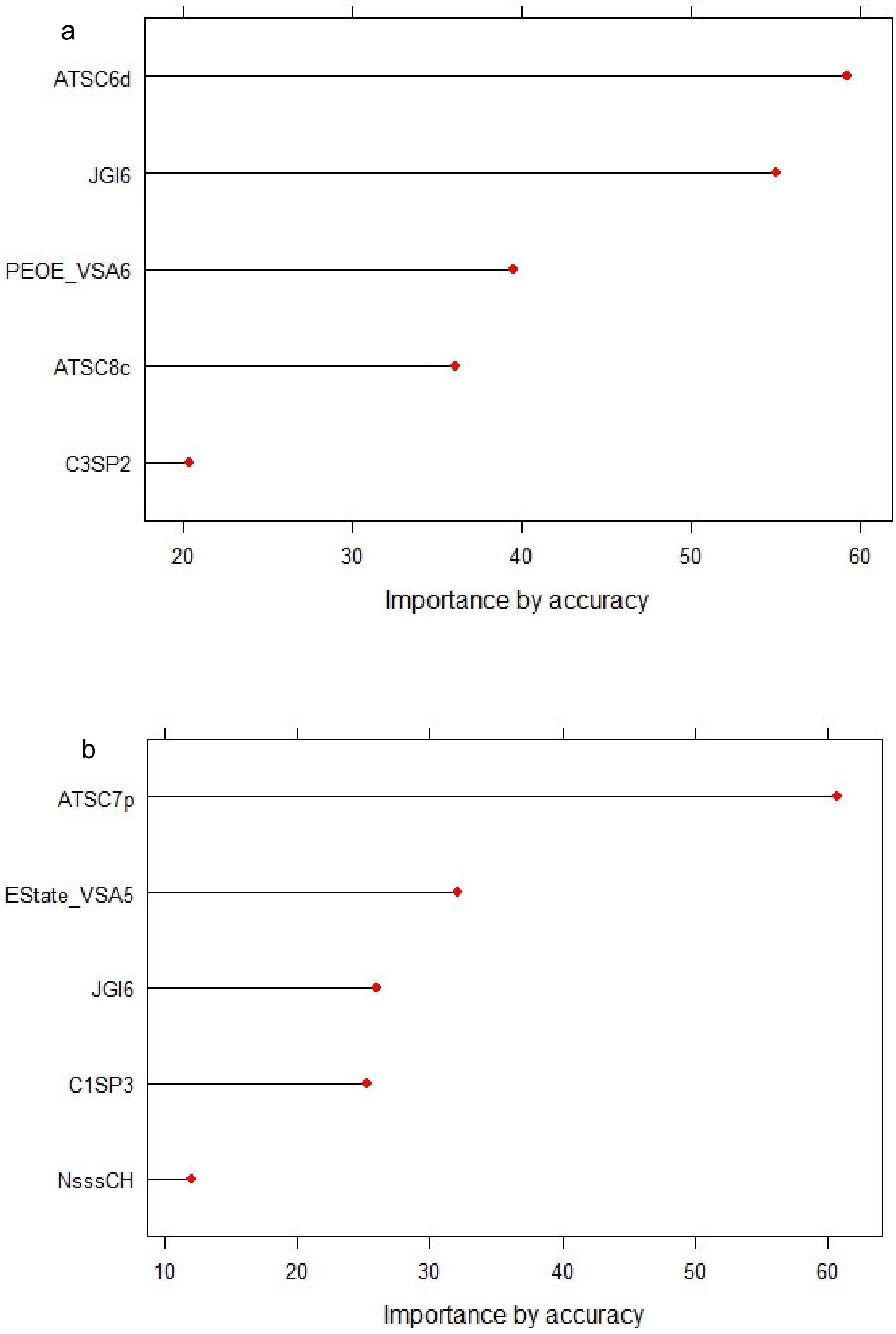

Figure S4: Importance of selected features based on accuracy for (a) OR1A1 and (b) OR2W1

Table S3: Description and significance of selected features (molecular descriptors)

Receptor	Feature	Description	Feature class	Significance
OR1A1	ATSC6d	Centered moreau-broto autocorrelation of lag 6 weighted by sigma electrons	2D-autocorrelation	Estimates log P-values
OR1A1	ATSC8c	Broto autocorrelation of lag 8 weighted by gasteiger charge		
OR2W1	ATSC7p	Centered moreau-broto autocorrelation of lag 7 weighted by polarizability		
OR1A1 OR2W1	JGI6	6-ordered mean topological charge	Topological	Contains structural information: size, symmetry, etc.
OR1A1	PEOE_VSA6	MOE charge VSA descriptor 6 (-0.10 <= x< -0.05	Surface area partial charge	Depicts surface electrostatistics characteristics
OR2W1	Estate_VSA5	Estate VSA descriptor 5 (1.17 <= x< 1.54		
OR1A1	C3SP2	SP2 carbon bound to 3 other carbons	Carbon types	Carbon connectivity characterization in terms of hybridization
OR2W1	C1SP3	SP3 carbon bound to1 other carbon		
OR2W1	Nsssch	Number of sssch	Electro-topological state indices	Hybridization states characteristics

Table S4: Statistical tests for the performance of classifiers. Best values are in bold with next best values in italics.

	RF		SVM		NB	
Dataset	p-value	kappa	p-value	kappa	p-value	kappa
OR1A1-training	<2e-16	0.99	<2e-16	0.78	<2e-16	0.55
OR1A1-testing	0.57	0.47	0.22	0.51	0.95	0.25
OR2W1-training	<2e-16	1	<2e-16	0.70	<2e-16	0.48
OR2W1-testing	0.04	0.56	0.77	0.25	0.77	0.27

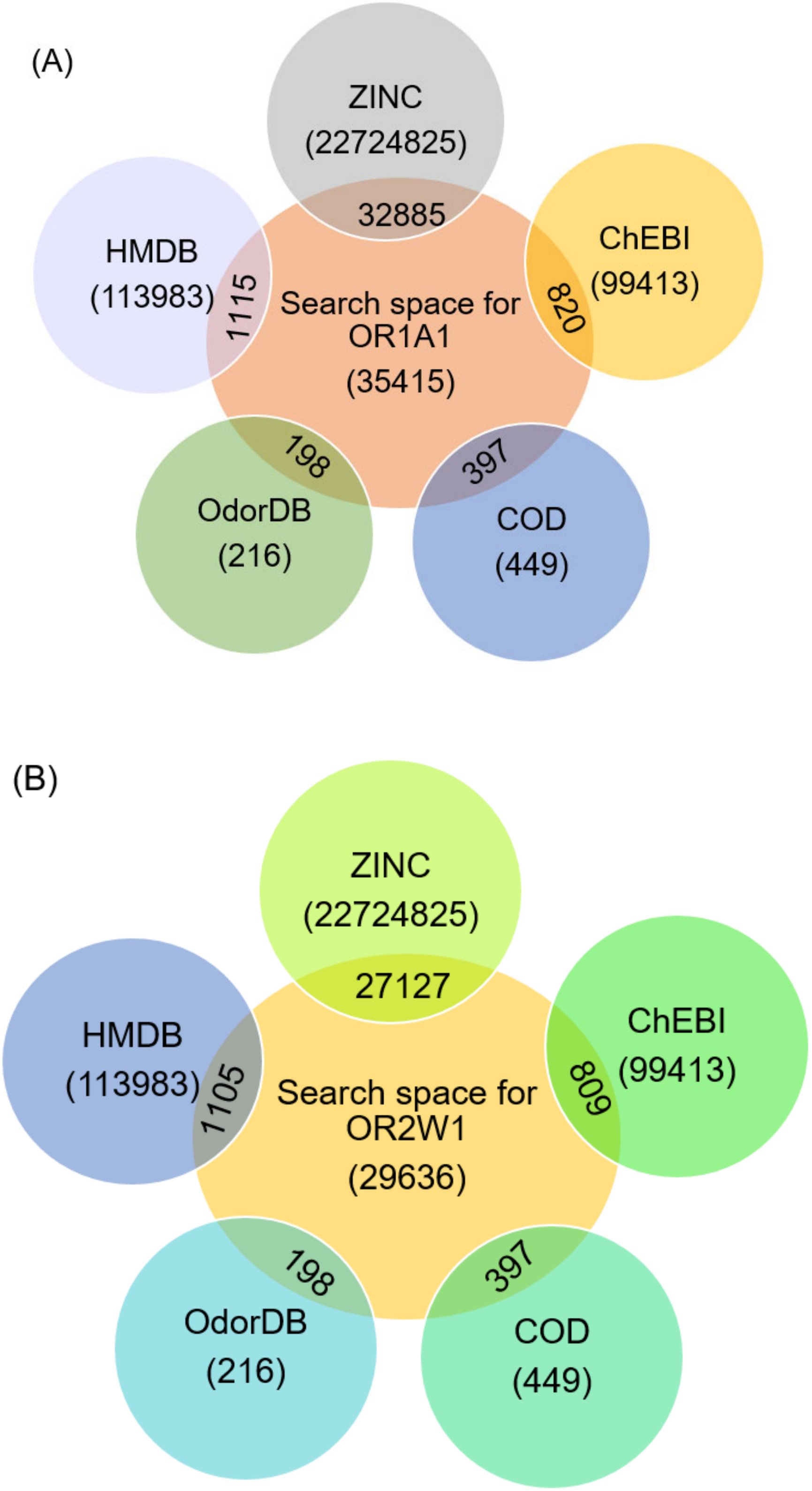

Figure S5: Search space statistics for (a) OR1A1 and (b) OR2W1

```
OR1A1           MRE-NNQSSTLEFILLGVTGQQEQEDFFYILFLFIYPITLIGNLLIVLAICSDVRLHNPMYFLLANLSLVDIFFSSVTIP 79
OR1A2           MKK-ENQSFNLDFILLGVTSQQEQNNVFFVIFLCIYPITLTGNLLIILAICADIRLHNPMYFLLANLSLVDIIFSSVTIP 79
OR1G1           MEG-KNLTSISECFLLGFSEQLEEQKPLFGSFLFMYLVTVAGNLLIILVIITDTQLHTPMYFFLANLSLADACFVSTTVP 79
OR2AG1          MEL-WNFTLGSGFILVGILNDSGSPELLCATITILYLLALISNGLLLLAITMEARLHMPMYLLLGQLSLMDLLFTSVVTP 79
OR2M3           MAR-ENSTFNSDFILLGIFNHSPTHTFLFFLVLAIFSVAFMGNSVMVLLIYLDTQLHTPMYLLLSQLSLMDLMLICTTVP 79
OR5AN1          MTGGGNITEITYFILLGFSDFPRIIKVLFTIFLVIYITSLAWNLSLIVLIRMDSHLHTPMYFFLSNLSFIDVCYISSTVP 80
OR7D4           MEA-ENLTELSKFLLLGLSDDPELQPVLFGLFLSMYLVTVLGNLLIILAVSSDSHLHTPMYFFLSNLSFVDICFISTTVP 79
OR51E2          MSS-CNFTH-ATFVLIGIPGLEKAHFWVGFPLLSMYVVAMFGNCIVVFIVRTERSLHAPMYLFLCMLAAIDLALSTSTMP 78
OR2W1           MDQ-SNYSSLHGFILLGFSNHPKMEMILSGVVAIFYLITLVGNTAIILASLLDSQLHTPMYFFLRNLSFLDLCFTTSIIP 79
                *    * :     .*:*.           .   .  ::  :.  *  ::.     :  ** ***::*  *:  *         *

OR1A1           KMLANHLLGSKSISFGGCLTQMYFMIALGNTDSYILAAMAYDRAVAISRPLHYTTIMSPRSCIWLIAGSWVIGNANALPH 159
OR1A2           KVLANHLLGSKFISFGGCLMQMYFMIALAKADSYTLAAMAYDRAVAISCPLHYTTIMSPRSCILLIAGSWVIGNTSALPH 159
OR1G1           KMLANIQIQSQAISYSGCLLQLYFFMLFVMLEAFLLAVMAYDCYVAICHPLHYILIMSPGLCIFLVSASWIMNALHSLLH 159
OR2AG1          KALADFLRRENTISFGGCALQMFLALTMGGAEDLLLAFMAYDRYVAICHPLTYMTLMSSRACWLMVATSWILASLSALIY 159
OR2M3           KMAFNYLSGSKSISMAGCATQIFFYTSLLGSECFLLAVMAYDRYTAICHPLRYTNLMSPKICGLMTAFSWILGSTDGIID 159
OR5AN1          KMLSNLLQEQQTITFVGCIIQYFIFSTMGLSESCLMTAMAYDRYAAICNPLLYSSIMSPTLCVWMVLGAYMTGLTASLFQ 160
OR7D4           KMLVSIQARSKDISYMGCLTQVYFLMMFAGMDTFLLAVMAYDRFVAICHPLHYTVIMNPCLCGLLVLASWFIIFWFSLVH 159
OR51E2          KILALFWFDSREISFEACLTQMFFIHALSAIESTILLAMAFDRYVAICHPLRHAAVLNNTVTAQIGIVAVVRGSLFFFPL 158
OR2W1           QMLVNLWGPDKTISYVGCIIQLYVYMWLGSVECLLLAVMSYDRFTAICKPLHYFVVMNPHLCLKMIIMIWSISLANSVVL 159
                :         .. *:  .*  * :.    :    :    :  *::*  .**.  ** :  ::.         :         .

OR1A1           TLLTASLSFCGNQEVANFYCDITPLLKLSCSDIHFHVKMMYLG-VGIFSVPLLCIIVSYIRVFSTVFQVPSTKGVLKAFS 238
OR1A2           TLLTASLSFCGNQEVANFYCDIMPLLKLSCSDVHFNVKMMYLG-VGVFSLPLLCIIVSYVQVFSTVFQVPSTKSLFKAFC 238
OR1G1           TLLMNSLSFCANHEIPHFFCDINPLLSLSCTDPFTNELVIFITGGLTGLICVLCLIISYTNVFSTILKIPSAQGKRKAFS 239
OR2AG1          TVYTMHYPFCRAQEIRHLLCEIPHLLKVACADTSRYELMVYVMGVTFLIPSLAAILASYTQILLTVLHMPSNEGRKKALV 239
OR2M3           VVATFSFSYCGSREIAHFFCDFPSLLILSCSDTSIFEKILFICCIVMIVFPVAIIIASYARVILAVIHMGSGEGRRKAFT 239
OR5AN1          IGALLQLHFCGSNVIRHFFCDMPQLLILSCTDTFFVQVMTAILTMFFGIASALVIMISYGYIGISIMKITSAKGRSKAFN 240
OR7D4           ILLMKRLTFSTGTEIPHFFCEPAQVLKVACSNTLLNNIVLYVATALLGVFPVAGILFSYSQIVSSLMGMSSTKGKYKAFS 239
OR51E2          PLLIKRLAFCHSNVLSHSYCVHQDVMKLAYADTLPNVVYGLTAILLVMGVDVMFISLSYFLIIRTVLQLPSKSERAKAFG 238
OR2W1           CTLTLNLPTCGNNILDHFLCELPALVKIACVDTTTVEMSVFALGIIIVLTPLILILISYGYIAKAVLRTKSKASQRKAMN 239
                          .    : :  *    :: ::  :                         :  **  :  :::    *     **:

OR1A1           TCGSHLTVVSLYYGTVMGTYFRPLTNYS---LKDAVITVMYTAVTPMLNPFIYSLRNRDMKAALRKLFNKRIS------- 308
OR1A2           TCGSHLTVVFLYYGTTMGMYFRPLTSYS---PKDAVITVMYVAVTPALNPFIYSLRNWDMKAALQKLFSKRIS------- 308
OR1G1           TCSSHLSVVSLFFGTSFCVDFSSPSTHS--AQKDTVASVMYTVVTPMLNPFIYSLRNQEIKSSLRKLIWVRKIHS----- 312
OR2AG1          TCSSHLTVVGMFYGAATFMYVLPSSFHS--TRQDNIISVFYTIVTPALNPLIYSLRNKEVMRALRRVLGKYMLPAHST-- 315
OR2M3           TCSSHLLVVGMYYGAALFMYIRPTSDRS--PTQDKMVSVFYTILTPMLNPLIYSLRNKEVTRAFMKILGKGKSG------ 311
OR5AN1          TCASHLTAVSLFYTSGIFVYLSSSSGGS--SSFDRFASVFYTVVIPMLNPLIYSLRNKEIKDALKRLQKRKC-------- 310
OR7D4           TCGSHLCVVSLFYGTGLGVYLSSAVTHS--SQSSSTASVMYAMVTPMLNPFIYSLRNKDVKGALERLLSRADSCP----- 312
OR51E2          TCVSHIGVVLAFYVPLIGLSVVHRFGNSLHPIVRVVMGDIYLLLPPVINPIIYGAKTKQIRTRVLAMFKISCDKDLQAVG 318
OR2W1           TCGSHLTVVSMFYGTIIYMYLQPGNRAS--KDQGKFLTLFYTVITPSLNPLIYTLRNKDMKDALKKLMRFHHKSTKIKRN 317
                ** **: .*   :: .      .        *            :*  : * :**:**  :. ::   .   :

OR1A1           --S 309
OR1A2           --S 309
OR1G1           --P 313
OR2AG1          --L 316
OR2M3           --E 312
OR5AN1          --C 311
OR7D4           --- 312
OR51E2          GK- 320
OR2W1           CKS 320
```

Figure S6: Alignment of olfactory receptor sequences with mutagenesis data and OR2W1. The predicted binding residues for OR2W1 and OR1A1 are highlighted in green, the mutagenesis data for OR1A1 in red, the consensus sites for OR1A1 i.e. sites that are predicted as well as experimentally known are highlighted in teal, OR1A2 in yellow, OR1G1 in blue, OR2AG1 in aqua, OR2M3 in pink, OR5AN1 in purple, OR7D4 in olive green, and OR51E2 in grey.

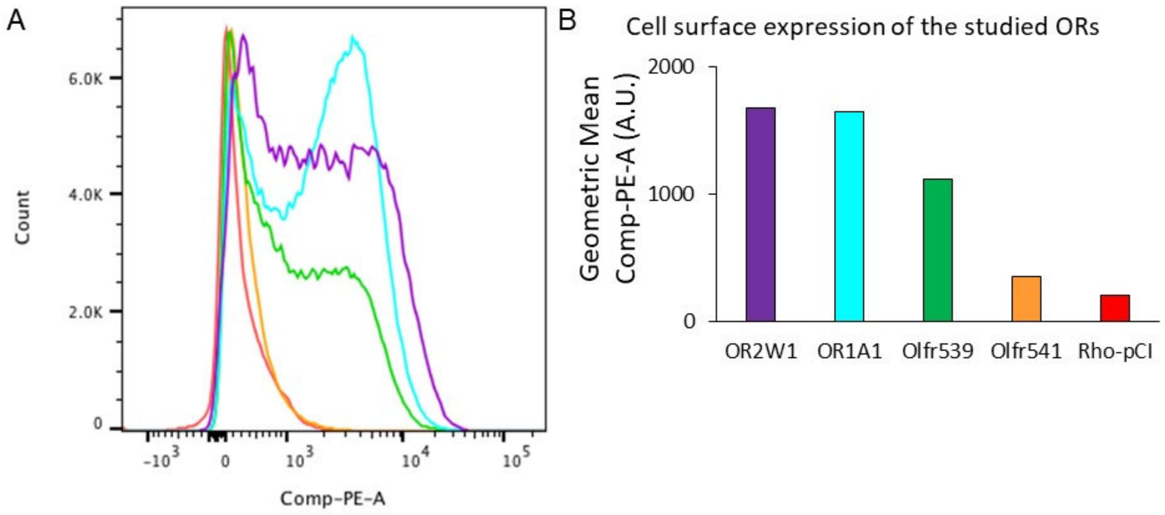

Sample Name	Geometric Mean : Comp-PE-A
OR2W1.fcs	1677
OR1A1.fcs	1647
Olfr539.fcs	1123
Olfr541.fcs	354
Rho-pCI.fcs	201

Figure S7: Cell surface expression of the studied ORs. A. Flow cytometry histogram showing the frequency of PE positive cells among the GFP positive, 7AAD negative, single and spheric cells. B. Geometric mean of the PE frequency for each ORs. Olfr539 and Olfr541 are positive and negative controls, respectively, of OR cell surface expression. Rho-pCI is the empty vector negative control. *In vitro* data is available from the Supplementary Data file (in Excel format).

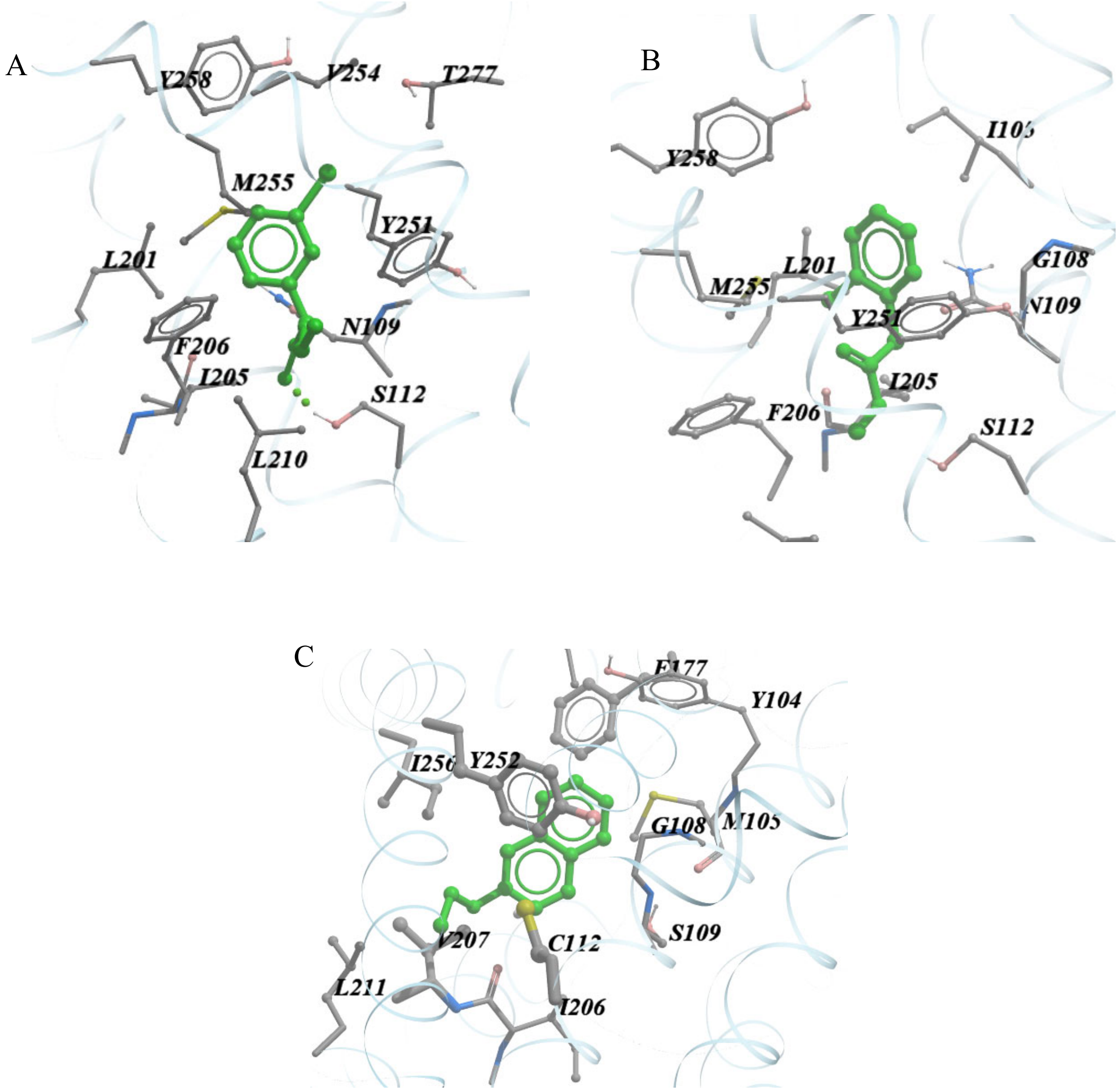

Figure S8: Binding mode of agonists and their cognate receptors. (A) Ethyl 2-(3-bromophenyl)acetate, (B) Methyl 2-(2-methylphenyl)acetate within OR1A1 binding pocket and (C) 2-ethoxynaphthalene within OR2W1 binding pocket. The agonists are shown in green colour.

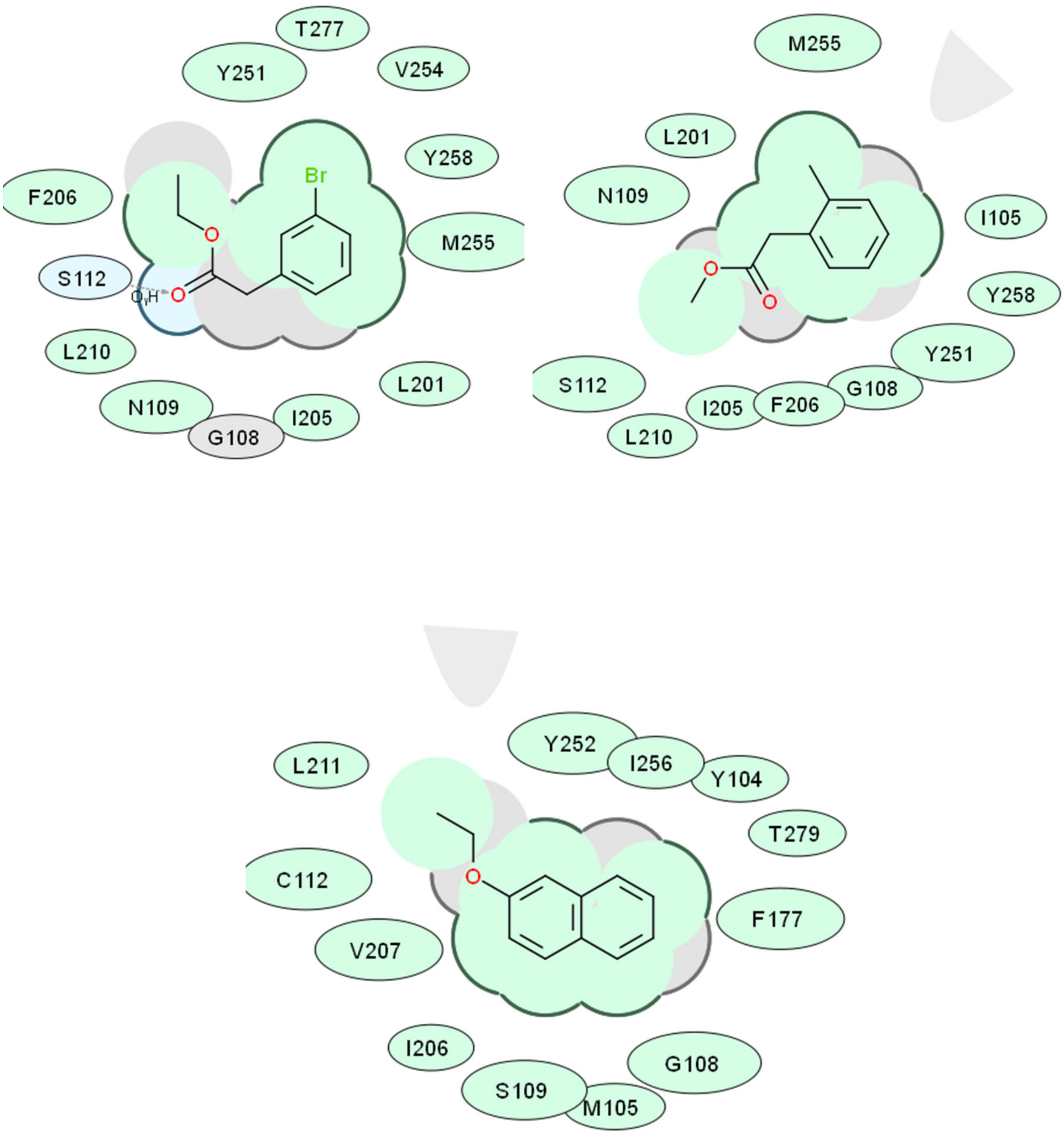

Figure S9: Interactions of agonists and their cognate receptors. (A) Ethyl 2-(3-bromophenyl) acetate, (B) Methyl 2-(2-methylphenyl)acetate with OR1A1 and (C) 2-ethoxynaphthalene with OR2W1: Green shading representing hydrophobic regions, gray shading represents wander waal interactions and gray parabolas represents surface accessible regions while blue shading represents hydrogen bond acceptor.

References

1. Schmiedeberg, K., E. Shirokova, H.P. Weber, B. Schilling, W. Meyerhof, and D. Krautwurst, *Structural determinants of odorant recognition by the human olfactory receptors OR1A1 and OR1A2.* J Struct Biol, 2007. **159**(3): p. 400-12.
2. Saito, H., Q. Chi, H. Zhuang, H. Matsunami, and J.D. Mainland, *Odor coding by a Mammalian receptor repertoire.* Sci Signal, 2009. **2**(60): p. ra9.
3. Geithe, C., F. Noe, J. Kreissl, and D. Krautwurst, *The Broadly Tuned Odorant Receptor OR1A1 is Highly Selective for 3-Methyl-2,4-nonanedione, a Key Food Odorant in Aged Wines, Tea, and Other Foods.* Chem Senses, 2017. **42**(3): p. 181-193.
4. Belloir, C., M.L. Miller-Leseigneur, F. Neiers, L. Briand, and A.M. Le Bon, *Biophysical and functional characterization of the human olfactory receptor OR1A1 expressed in a mammalian inducible cell line.* Protein Expr Purif, 2017. **129**: p. 31-43.
5. Mainland, J.D., A. Keller, Y.R. Li, T. Zhou, C. Trimmer, L.L. Snyder, A.H. Moberly, K.A. Adipietro, W.L. Liu, H. Zhuang, S. Zhan, S.S. Lee, A. Lin, and H. Matsunami, *The missense of smell: functional variability in the human odorant receptor repertoire.* Nat Neurosci, 2014. **17**(1): p. 114-20.
6. Sato-Akuhara, N., N. Horio, A. Kato-Namba, K. Yoshikawa, Y. Niimura, S. Ihara, M. Shirasu, and K. Touhara, *Ligand Specificity and Evolution of Mammalian Musk Odor Receptors: Effect of Single Receptor Deletion on Odor Detection.* J Neurosci, 2016. **36**(16): p. 4482-91.
7. Bushdid, C., C.A. de March, S. Fiorucci, H. Matsunami, and J. Golebiowski, *Agonists of G-Protein-Coupled Odorant Receptors Are Predicted from Chemical Features.* J Phys Chem Lett, 2018. **9**(9): p. 2235-2240.
8. Adipietro, K.A., J.D. Mainland, and H. Matsunami, *Functional evolution of mammalian odorant receptors.* PLoS Genet, 2012. **8**(7): p. e1002821.
9. Braun, T., P. Voland, L. Kunz, C. Prinz, and M. Gratzl, *Enterochromaffin cells of the human gut: sensors for spices and odorants.* Gastroenterology, 2007. **132**(5): p. 1890-901.
10. Li, S., L. Ahmed, R. Zhang, Y. Pan, H. Matsunami, J.L. Burger, E. Block, V.S. Batista, and H. Zhuang, *Smelling Sulfur: Copper and Silver Regulate the Response of Human Odorant Receptor OR2T11 to Low-Molecular-Weight Thiols.* J Am Chem Soc, 2016. **138**(40): p. 13281-13288.
11. Audouze, K., A. Tromelin, A.M. Le Bon, C. Belloir, R.K. Petersen, K. Kristiansen, S. Brunak, and O. Taboureau, *Identification of odorant-receptor interactions by global mapping of the human odorome.* PLoS One, 2014. **9**(4): p. e93037.
12. McRae, J.F., J.D. Mainland, S.R. Jaeger, K.A. Adipietro, H. Matsunami, and R.D. Newcomb, *Genetic variation in the odorant receptor OR2J3 is associated with the ability to detect the "grassy" smelling odor, cis-3-hexen-1-ol.* Chem Senses, 2012. **37**(7): p. 585-93.
